# Draft Genome Sequence of *Staphylococcus aureus* 1608, a Strain That Caused Toxic Mastitis in Twin Cows

**DOI:** 10.1128/genomeA.01438-16

**Published:** 2017-01-05

**Authors:** Marc J. A. Stevens, Roger Stephan, Sophia Johler

**Affiliations:** a Laboratory of Food Biotechnology, Institute of Food, Nutrition and Health, ETH Zurich, Zurich, Switzerland; b Institute for Food Safety and Hygiene, Vetsuisse Faculty, University of Zurich, Zurich, Switzerland

## Abstract

*Staphylococcus aureus* 1608 is a strain that caused a lethal mastitis in cows. Here, the draft genome sequence of the strain is presented.

## GENOME ANNOUNCEMENT

Bovine mastitis results in substantial financial losses for the dairy industry. *Staphylococcus aureus* can cause subclinical, acute, or chronic bovine mastitis and has recently been linked to “toxic mastitis,” which typically combines mastitis symptoms and clinical signs of toxemia (1–3). In this study, we present the complete genome sequence of *S. aureus* 1608. A case report detailing the clinical findings and DNA microarray data has already been published (4). On 3 December 2013, the strain was isolated from a mastitis milk sample of a Simmental cow in Switzerland. The cow suffered from characteristic signs of toxic mastitis, including anorexia, pyrexia (41.5°C), an elevated heart rate, and a swollen hardened mammary gland exhibiting brownish secretions. Due to rapid deterioration of the clinical state of the animal, it had to be euthanized within 48 h after presentation. *S. aureus* strain 1608 was assigned to *agr* type I, capsule type 5, and clonal complex 97 (CC97) (4).

The genome of strain 1608 was sequenced using Ilumina MiSeq 125-bp paired-end sequence technology. The raw reads were paired in the CLC Genomics Workbench 8.0, resulting in 1,411,718 reads with a mean size of 250 bp. The reads were assembled in CLC Genomics Workbench 8.0 using standard settings, which resulted in 34 contigs with sizes between 3,142 and 318,947 bp.

The genome was estimated at 2.73 Mbp and annotated by the NCBI Prokaryotic Genome Annotation Pipeline. It contains 2,772 protein-coding sequences, and the G+C content of the genome is 32.1%. The genome content of *Staphylococcus aureus* 1608 could contribute to the identification of factors causing toxic mastitis in cattle.

### Accession number(s).

Sequence and annotation data of *S. aureus* strain 1608 were deposited in the GenBank database with the accession number MKYV00000000.
